# Increasing tPA Activity in Astrocytes Induced by Multipotent Mesenchymal Stromal Cells Facilitate Neurite Outgrowth after Stroke in the Mouse

**DOI:** 10.1371/journal.pone.0009027

**Published:** 2010-02-03

**Authors:** Hongqi Xin, Yi Li, Li Hong Shen, Xianshuang Liu, Xinli Wang, Jing Zhang, Siamak Pourabdollah-Nejad D, Chunling Zhang, Li Zhang, Hao Jiang, Zheng Gang Zhang, Michael Chopp

**Affiliations:** 1 Department of Neurology, Henry Ford Health System, Detroit, Michigan, United States of America; 2 Department of Physics, Oakland University, Rochester, Michigan, United States of America; University of São Paulo, Brazil

## Abstract

We demonstrate that tissue plasminogen activator (tPA) and its inhibitors contribute to neurite outgrowth in the central nervous system (CNS) after treatment of stroke with multipotent mesenchymal stromal cells (MSCs). *In vivo*, administration of MSCs to mice subjected to middle cerebral artery occlusion (MCAo) significantly increased activation of tPA and downregulated PAI-1 levels in the ischemic boundary zone (IBZ) compared with control PBS treated mice, concurrently with increases of myelinated axons and synaptophysin. *In vitro*, MSCs significantly increased tPA levels and concomitantly reduced plasminogen activator inhibitor 1 (PAI-1) expression in astrocytes under normal and oxygen and glucose deprivation (OGD) conditions. ELISA analysis of conditioned medium revealed that MSCs stimulated astrocytes to secrete tPA. When primary cortical neurons were cultured in the conditioned medium from MSC co-cultured astrocytes, these neurons exhibited a significant increase in neurite outgrowth compared to conditioned medium from astrocytes alone. Blockage of tPA with a neutralizing antibody or knock-down of tPA with siRNA significantly attenuated the effect of the conditioned medium on neurite outgrowth. Addition of recombinant human tPA into cortical neuronal cultures also substantially enhanced neurite outgrowth. Collectively, these *in vivo* and *in vitro* data suggest that the MSC mediated increased activation of tPA in astrocytes promotes neurite outgrowth after stroke.

## Introduction

Cultured medium from multipotent mesenchymal stromal cells (MSCs) increases neurite outgrowth in cultured neurons [1], and MSC treatment of stroke enhances functional recovery and increases neurite outgrowth in rodents [2], [3]. MSCs secrete and stimulate parenchymal cell production of bioreactive factors in brain after stroke [4]–[7]. We therefore sought to identify the key restorative factors that promote MSC stimulated neurite outgrowth.

The plasminogen activator (PA)/plasmin system is a major proteolytic system in the adult central nervous system (CNS) [8]–[11]. With specific inhibitors, i.e., plasminogen activator inhibitor (PAI)-1 (encoded by *serpine 1* gene, secreted by neurons and active astrocytes) and neuroserpin (encoded by *serpini 1* gene, secreted by neurons) [12]–[14], the activity of the PA/plasmin system is in equilibrium in the mammalian brain. The PA/plasmin system and its inhibitors participate in a number of physiological and pathological events in the CNS [15]–[17], and facilitate neurite outgrowth and sustain synaptic plasticity via interaction with extracellular matrix proteoglycans [18]–[20].

In brain, tissue plasminogen activator (tPA) expression in astrocytes is the primary source of plasminogen activator and PAI-1 is the dominant inhibitor of tPA [21]. Gene array analysis of primary astrocyte cultures derived from wild-type (WT) and glial fibrillary acidic protein (GFAP)/vimentin (Vim) double knock-out mice reveal that only the PAI-1 gene, out of 1200 genes measured was downregulated by threefold or higher in the knock-out animals [22]. MSCs modify ischemia-induced astrocytic activation and reduce GFAP expression in astrocytes in vitro [23] and significantly reduce the thickness of the scar wall in vivo [3], [24]. Therefore, we hypothesize that MSCs decrease PAI-1 expression and stimulate tPA after ischemia and thereby promote neurite remodeling.

In this study, we measured tPA/PAI-1 expression and tPA activity in astrocytes cultured under normal and oxygen and glucose deprivation (OGD) conditions and co-cultured with or without MSCs as an in vitro ischemia model. To test the effects of tPA/PAI-1 in astrocytes on neurite outgrowth, conditioned media from cultured astrocytes were added to primary cultured cortical neurons. In addition, mice subjected to middle cerebral artery occlusion (MCAo) were employed to test for tPA activity and neurite outgrowth in vivo.

## Results

### MSC Co-Culture Alters tPA and PAI-1 Expression in Normal and OGD Astrocytes

**Figure 1:**
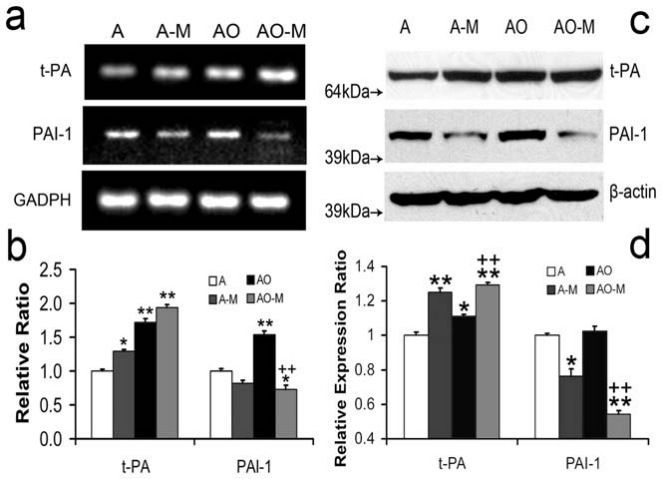
tPA and PAI-1 mRNA and protein levels in treated astrocytes. qRT-PCR shows mRNA levels of tPA and PAI-1 in normal cultured astrocytes (A), astrocytes co-cultured with MSCs (A-M), OGD astrocytes (AO) and OGD astrocytes co-cultured with MSCs (AO-M) (a). OGD treatment significantly increased tPA and PAI mRNA levels in astrocytes. MSC co-culture significantly increased tPA mRNA level in both normal and OGD astrocytes whereas MSC co-culture significantly decreased PAI-1 mRNA level (b) in OGD astrocytes. Western blot shows protein levels of tPA and PAI-1 in normal cultured astrocytes (A), astrocytes co-cultured with MSCs (A-M), OGD astrocytes (AO) and OGD astrocytes co-cultured with MSCs (AO-M) (c). OGD treatment increased tPA and PAI protein level and co-culture MSCs increased tPA protein level whereas MSCs decreased PAI-1 protein level (d). *P<0.05, **P<0.01, compared with group A; ++P<0.01, compared with group AO.

qRT-PCR was employed to measure tPA and PAI-1 mRNA in cultured astrocytes responding to OGD and MSC co-culture. Fig. 1a, b shows that normal cultured astrocytes express tPA and PAI-1 mRNA. tPA and PAI-1 mRNA levels were significantly increased in astrocytes subjected to OGD compared to normal astrocytes, respectively. MSC co-culture significantly increased the tPA mRNA levels in both normal and OGD astrocytes, whereas MSCs significantly decreased the PAI-1 mRNA level in OGD astrocytes (1b) compared to normal and OGD astrocytes without MSC co-culture, respectively.

Western blot was employed to measure the tPA and PAI-1 protein levels in cultured astrocytes in response to OGD and MSC co-culture (Fig. 1c, d). OGD treatment significantly increased tPA and slightly increased PAI-1 protein levels in astrocytes. MSC co-culture significantly increased tPA and decreased the PAI-1 protein levels in normal and OGD astrocytes compared to normal and OGD astrocytes without MSC co-culture, respectively (1d).

### MSC Co-Culture Alters tPA Level and Activity in Conditioned Medium

When tPA is bound with PAI-1 or its other inhibitors, tPA is inactive [25]; conversely, tPA is active when unbound. Active mouse tPA binds to the biotinylated human PAI-1 coated on a microtiter, and an ELISA kit can be used to measure the active tPA in conditioned media. The total tPA protein and active tPA in various conditioned media were measured with ELISA kits (Table 1). Normal cultured astrocytes secreted tPA at a concentration of 1.27±0.02 ng/mL, and normal astrocytes co-cultured with MSCs significantly (p<0.05) increased the tPA concentration to 1.32±0.01 ng/mL. In OGD astrocytes, MSC co-culture increased the tPA concentration to 2.24±0.08 ng/mL compared to OGD astrocytes without MSC co-culture (2.14±0.14 ng/mL). tPA concentrations were significantly increased in OGD astrocytes with or without MSC co-culture compared with normal cultured astrocytes (p<0.01), respectively. The active tPA concentration in normal cultured astrocyte medium was 0.31±0.01 ng/mL, and MSCs significantly increased the active tPA concentration in normal astrocytes to 0.49±0.02 ng/mL. MSC co-culture significantly increased the active tPA concentration to 0.36±0.02 ng/mL in OGD astrocytes compared with OGD astrocytes without MSC co-culture (0.24±0.03 ng/mL).

**Table 1:** Total tPA protein and active tPA concentration in various conditioned media (n = 6/group).

Groups	Total tPA concentration (ng/mL)	Active tPA concentration (ng/mL)
**A**	1.27±0.02	0.31±0.01
**A-M**	1.32±0.01*	0.49±0.02**
**AO**	2.14±0.14**	0.24±0.03*
**AO-M**	2.24±0.08**	0.36±0.02##

A: medium from normal cultured astrocytes; A-M: medium from normal astrocytes co-cultured with MSCs; AO: medium from OGD astrocytes; AO-M: medium from OGD astrocytes co-cultured with MSCs.

*P<0.05.

**P<0.01 compared with group A.

## P<0.01 compared with group AO.

**Figure 2:**
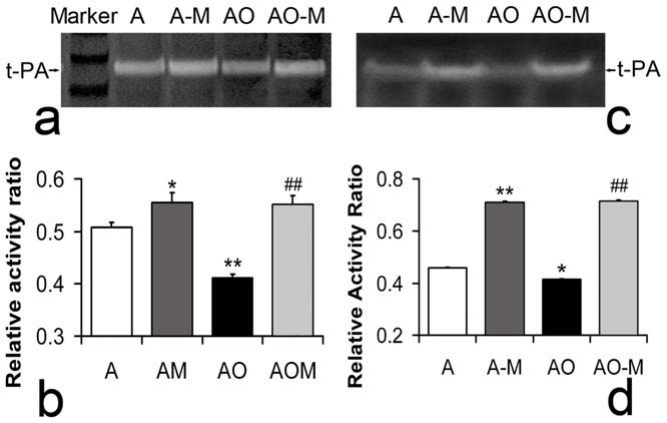
tPA activity of astrocyte lysates and conditional media. Zymography (a, b) shows that MSCs significantly increased tPA activity in normal cultured astrocytes and in OGD treated astrocytes (c), and similar results were obtained using the conditioned media harvested from the concomitant groups (d). Marker: prestained protein marker; A: normal cultured astrocytes; A-M: normal astrocytes co-cultured with MSCs; AO: OGD astrocytes; AO-M: OGD astrocytes co-cultured with MSCs. *P<0.05, **P<0.01, compared with group A; ##P<0.01, compared with group AO.

Only active tPA can perform proteolytic function; we therefore used the direct casein zymography assay to visualize and measure tPA activity in cultured astrocytes and conditioned media under normal and OGD conditions and to determine whether tPA activity is modified by MSC co-culture. Fig. 2a, 2b show that OGD treatment significantly decreased the tPA activity in astrocytes. MSCs significantly increased this activity both in normal astrocytes and in OGD treated astrocytes. Similar results were obtained in the conditioned media harvested from the concomitant groups of cultured astrocytes (Fig. 2c, 2d).

### MSCs Increase Neurite Outgrowth of Cortical Neurons via Activated tPA

To test whether tPA in conditioned media affects neurite outgrowth, a primary culture cortical neuronal system was used. Primary cultured cortical neurons were treated with various conditioned media for 4 days. A tPA neutralizing antibody was used to reduce the effects of tPA, and rh-tPA was used as a positive control. The normal control group was cultured with neurobasal culture medium.

**Figure 3:**
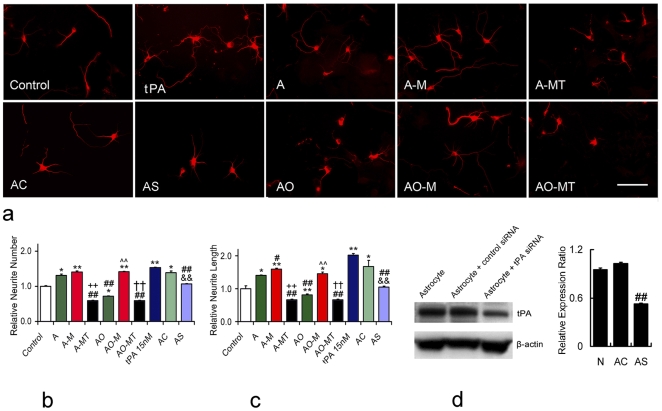
Neurite outgrowth in primary cultured cortical neuron after conditional media treatment. Fluorescence microscopy (a) shows cortical neurite outgrowth. Control: primary cultured cortical neurons with neurobasal medium; medium from normal cultured astrocytes (A) increased neurite number and total length compared to those in control group; medium from OGD astrocytes (AO) significantly decreased neurite number and total length; media from normal astrocytes co-cultured with MSCs (A-M) and OGD astrocytes co-cultured with MSCs (AO-M) increased neurite total length compared to that in A and AO groups, respectively, and increased neurite number in AO-M group compared with AO group. tPA neutralizing antibody specifically antagonized tPA effects of AM and AO-M groups in neurite number and total length (b, c). Western blot shows that tPA expression in astrocytes was substantially down regulated by tPA siRNA (d). Medium from tPA knock-down astrocytes significantly reduced the neurite number and total length of cultured neurons compared with that from normal astrocytes or negative control siRNA transfected astrocytes (b, c). A-MT: medium from normal astrocytes co-cultured with MSCs, t-PA neutralizing antibody presented; AO-MT: medium from OGD astrocytes co-cultured with MSCs, t-PA neutralizing antibody presented; t-PA: 15nM rh-t-PA alone; AC: medium from astrocytes transfected with negative control siRNA; AS: medium from astrocytes transfected with tPA siRNA. Scale bars = 50 µm. *P<0.05, ** P<0.01, compared with control group; #P<0.05, ##P<0.01, compared with A group; ++P<0.01, compared with A-M group; ∧∧P<0.01, compared with AO group; ††P<0.01, compared with AO-M group; &&P<0.01, compared with AC group. Data are presented as Mean±SE, (neurons n = 50/group, Adjusted p-value = 0.0042).

Fig. 3a shows the typical morphology of cultured neurons treated with conditioned media. rh-tPA treatment significantly increased the neurite branch number and total neurite length of cultured neurons. Compared to neurobasal medium cultured neurons, the neurite branch number and total length were significantly increased when treated with the normal cultured astrocyte conditioned medium. The OGD astrocyte medium significantly decreased neurite branch number and total length. Co-culture with MSCs significantly increased neurite branch number and total length both of neurons cultured with normal cultured astrocyte medium and with OGD astrocyte medium, respectively. The tPA neutralizing antibody sharply counteracted the effects of MSC co-culture media on neurite number and total length, and neurite branch number and length were significantly reduced compared with normal control group (Fig. 3b, 3c). To verify the neurite outgrowth promoting effect of tPA secreted by astrocytes, the siRNA technique was used to knock-down the tPA expression in astrocytes. Fig. 3d shows the tPA level was substantially down regulated by transfecting astrocytes with tPA siRNA. Medium from tPA knock-down astrocytes significantly reduced the neurite number and total length of cultured neurons compared with medium from normal astrocytes or negative control siRNA transfected astrocytes (Fig. 3b, 3c).

### Endogenous tPA Expression Level and Activity in Mouse Brain after Stroke with and without MSC Treatment

**Figure 4:**
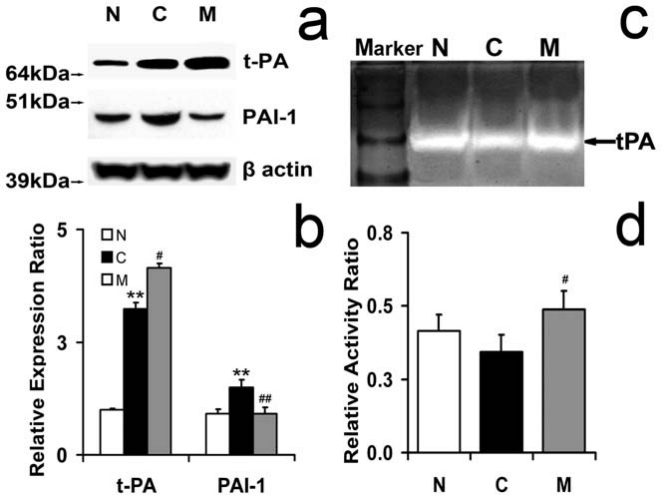
tPA and PAI-1 levels and tPA activity in mice subjected to MCAo with or without MSC treatment. Western blot shows tPA protein level (a) and zymography shows tPA activity (b) in MCAo mice with or without MSC treatment. tPA and PAI-1 expression was significantly increased in the IBZ of mice subjected to MCAo compared with normal mice. tPA expression was significantly increased and PAI-1 expression was significantly decreased in the IBZ of MCAo mice after MSC treatment compared with MCAo alone mice (c). MSC treatment significantly increased the activity of tPA in the IBZ compared with MCAo alone mice (d). N: normal mouse brain tissue; C: IBZ tissue from MCAo mice; M: IBZ tissue from MCAo mice after MSC treatment. **P<0.01, compared with normal mice; #P<0.05, ##P<0.01, compared with control MCAo mice.

Stroke significantly increased expression of tPA and PAI-1 in the ischemic hemisphere compared to non-ischemic brain (Fig. 4a, 4b). Treatment of stroke with MSCs significantly increased tPA expression and decreased PAI-1 expression compared with PBS treatment. tPA activity in the MCAo control brain extract and in the MCAo MSC treated brain extract was measured using zymography. Fig. 4c, 4d show that MSC treatment significantly increased the activity of tPA compared to MCAo alone mice at 14 days after stroke.

### tPA and PAI-1 mRNA Levels in Astrocytes Located within the Ischemic Boundary Zone (IBZ) Respond to MSCs

**Figure 5:**
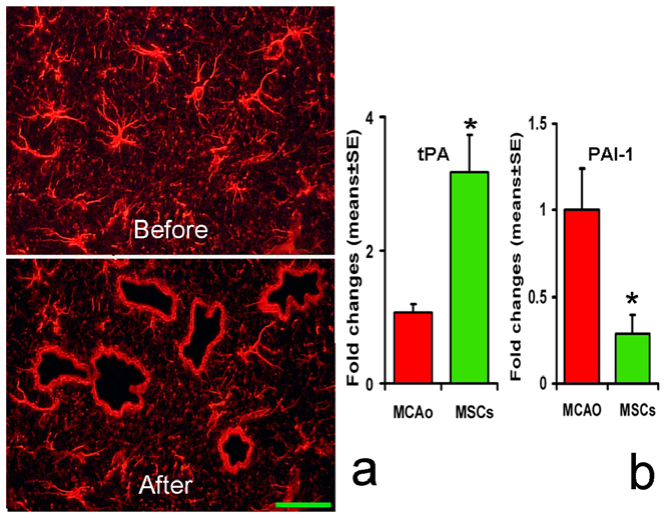
tPA and PAI-1 level in MCAo mouse IBZ astrocytes respond to MSC. (5a) presents the individual IBZ astrocytes dissected using LCM (before and after dissection), and tPA and PAI-1 mRNA level in these astrocytes with or without MSC treatment are shown in (b). MSC treatment significantly increased tPA mRNA level and concomitantly decreased PAI-1 mRNA level. Scale bars = 50 µm. *P<0.05 compared with MCAo mice.

We employed laser capture microdissection (LCM) combined with RT-PCR to measure tPA and PAI-1 mRNA levels in astrocytes in the IBZ (Fig. 5a) after stroke with or without MSC treatment. Fig. 5b shows that MSC treatment significantly increased the tPA mRNA level and concomitantly decreased the PAI-1 mRNA level. These data indicate that astrocytes in the IBZ respond to MSC treatment and increase tPA and concomitantly decrease PAI-1 gene expression.

### MSC Treatment Increases Axonal Fiber and Synaptic Regeneration in the IBZ

**Figure 6:**
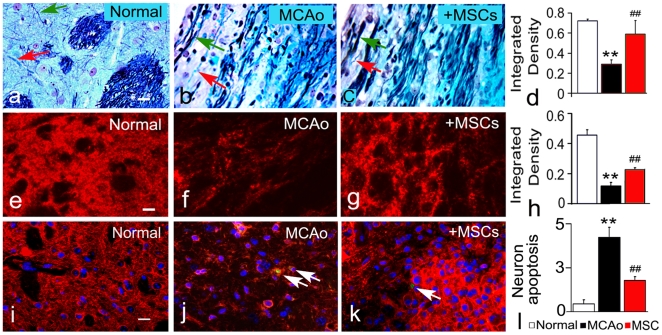
MSCs promote neurite outgrowth, synaptic plasticity and cell survival. Double staining (Bielshowsky, black for axons, indicated by green arrow; Luxol fast blue for myelin, indicated by red arrow.) shows axonal and myelin fibers in the striatum (b–c) along the IBZ after MCAo compared to normal brain (a). The integrated density (indicated the axonal and myelin fibers number) of white matter bundles in IBZ of striatum was decreased compared with that in normal brain, The axonal fibers and myelin of the striatum were enhanced by MSCs (c, d, Adjusted p-value = 0.0167). Immunofluorescent staining (e–g) shows synaptic regeneration (indicated by synaptophysin). Synaptophysin expression significantly increased in MSC treated rats (g, h, Adjusted p-value = 0.025). Apoptosis neuron (indicated by white arrow) is shown with double staining with TUNEL and MAP 2 (i–k), MSCs decreased the apoptosis neuron number (k, l, Adjusted p-value = 0.01667). Scale bars = 25 µm. **P<0.01, compared with normal mice; ##P<0.01, compared with control MCAo alone mice.

Double staining of Bielshowsky silver and Luxol fast blue can identify axonal fibers and myelin, respectively, in the white matter of the brain [26]. The ischemic attack severely damaged white matter bundles in the core lesion area and axon-myelin bundles were altered and appeared disorganized in the IBZ (top-left in Fig. 6b, 6c) of the striatum compared to normal mice (Fig. 6a). Axonal fiber density in the IBZ of the striatum was significantly increased after MSC treatment (Fig. 6c, 6d) compared with MCAo control animals (Fig. 6b, 6d).

Synaptophysin is an indicator of presynaptic plasticity and synaptogenesis [27]. Stroke decreased synaptophysin expression in the IBZ of the striatum (Fig. 6f) compared to the normal mice (Fig. 6e). MSC treatment significantly increased synaptophysin expression (Fig. 6g, 6h) compared with MCAo control mice (Fig. 6f, 6h), suggesting that MSC treatment increases synaptic regeneration after MCAo.

To identify the effects of MSCs on neuronal apoptosis, we performed double staining with TUNEL and MAP 2. At 14 days after stroke, only few scattered apoptotic neurons were evident, and MSCs decreased the number of apoptotic neurons compared to MCAo control animals (Fig. 6i–6l). These data suggest that, although we cannot exclude a contribution of MSC induced neuroprotection to the MSC mediated increase in axonal fiber density and synaptophysin expression, the reduction in apoptosis likely plays a minor role in the observed white matter remodeling.

## Discussion

MSCs are localized to the boundary region of the ischemic infarct in rodents [28]–[32], MSCs improve recovery from stroke in mice and rats directly by secreting soluble factors [5], [6] and indirectly by stimulating parenchymal cells of the stroke brain to secrete bioactive factors [4], [7], [33]–[37], which induce neurogenesis, angiogenesis, white matter change, synaptogenesis and reduce apoptosis [4], [7], [38]–[41]. In this study, we show for the first time that MSCs also enhance neurite outgrowth, which benefits brain recovery after stroke, by concomitantly decreasing the expression of the tPA inhibitor PAI-1 and increasing the activity of tPA in astrocytes in the peri-infarct area of ischemic brain. Thus, the modulation of tPA activity by MSCs likely promotes neurite remodeling and thereby may improve functional outcome after stroke.

tPA, which influences neurite outgrowth, is expressed by many types of neural cells in the developing brain, including astrocytes [42]. Treatment of primary cortical neurons with an antibody against tPA significantly reduced neurite outgrowth and branch numbers compared to control neurons without tPA treatment. Using LCM, we have demonstrated that astrocytes in the IBZ respond to MSC treatment by increasing tPA activity (i.e. increase of tPA level with a concomitant decrease of PAI-1 level). We do not exclude a contribution from other parenchymal cells to the MSC enhanced tPA activity; however, the high concentration of astrocytes in the IBZ and the predominance of numbers astrocytes in parenchymal tissue, suggest that the astrocyte is a robust contributor to the MSC mediated tPA activity and subsequent functional recovery. This is the first study to suggest that tPA activity enhanced by astrocytes contributes to the therapeutic benefits of a cell-based therapy.

Extracellular matrix (ECM) degradation is needed for neurite outgrowth and the remodeling of crossing axonal fibers [18]. The plasmin system plays an active role in tissue remodeling. Plasmin degrades the ECM, directly by removing glycoproteins from the ECM [43] and it releases axonal guidance molecules from the extracellular matrix [19]. tPA has multifaceted effects on tissue; it interacts with parenchymal cells through proteolytic plasminogen or non-plasminogen pathways [44]–[47]. The proteolytic plasminogen/plasmin function of tPA cleaves the precursor forms of neurotrophins to the active forms of these trophic factors, e.g. proteolytic activity by tPA converts proBDNF and proNGF in the ECM to active trophic factors, respectively [48]–[50]. These trophic factors promote neurite remodeling [51]–[55]. The plasmin dependent pathway has multiple roles in addition to the cleavage of pro-neurotrophins into the active form [56]. Plasmin also activates the N-methyl-D-Aspartate receptor (NMDAR) which can subsequently enhance neurite remodeling [57]. Moreover, recent studies report that the NMDAR can act independently of the plasminogen pathway and stimulate nitric oxide synthase (NOS), which increases nitric oxide (NO) and subsequently increases cyclic guanosine monophosphate (cGMP) which can foster neurite remodeling [58]. Similarly, the low-density lipoprotein receptor pathway (LDLR), has been shown to mediate enhancement of NMDAR function by tPA [59]. In addition to the PA/plasmin system, tPA interacts with other effectors such as low-density lipoprotein receptor-related protein (LRP) [60], [61] and latent platelet-derived growth factor-CC (PDGF-CC) [62], [63] which may subsequently influence the brain ECM remodeling and neurite outgrowth. tPA converts PDGF-CC to an active form PDGF-C, which is expressed in embryonic and adult mouse brain and contributes to brain remodeling and spinal cord development [64]. MSC treatment increases tPA activity in astrocytes and thereby promotes white matter remodeling which likely contributes via multiple pathways to recovery of neurological function after stroke.

As a member of the serpine gene family, PAI-1 is the major inhibitor of tPA [21], and is largely produced by reactive astrocytes in the CNS after stroke [65], [66]. PAI-1 plays an important role in the process of peripheral tissue remodeling and fibrinolysis through inhibition of plasmin-dependent ECM degradation [67]. Hypoxia and many growth factors, including transforming growth factor beta (TGFβ) and tumor necrosis factor alpha (TNF-α), as well as other chemicals/agents, induce PAI-1 expression in cultured cells and in vivo [68]. The concomitant MSC induced reduction of PAI-1 and increase of tPA in astrocytes of the IBZ thereby amplify tPA activity which contributes to neurite outgrowth.

Both growth promoting and growth-inhibitory molecules are upregulated within the peri-infarct region of the brain early after stroke [69]. tPA expression as well as its inhibitors PAI-1 and neuroserpin are up-regulated in the acute stage of cerebral ischemia and hemorrhage [13], [70], [71]. An increase of parenchymal cell expression of tPA early after stroke may be a negative factor for neural cell survival and for sustaining blood-brain barrier (BBB) integrity. Elevated PAI-1 expression inhibits tPA activity and reduces injury from stroke [72], [73]. In addition to its damaging role, the tPA/plasmin proteolytic system provides benefits by facilitating neurite outgrowth and pathfinding [10], [11]. Our in vitro studies which mimic ischemic conditions show increased tPA and PAI-1 expression in OGD astrocytes, and that MSC-astrocyte co-culture concomitantly decreased the PAI-1 expression and increased tPA activity in astrocytes. In vivo, treatment of MCAo with MSCs increased tPA activity, and this increase likely contributed to the observed neurite outgrowth and synaptic plasticity. Our data provide a new insight, that MSC cell based therapy for stroke promotes neurite outgrowth, axonal regeneration and synaptic plasticity via the astrocytic tPA system.

The roles that reactive astrocytes take after brain injury are multi varied [74]–[76]. Astrocytes promote or inhibit axonal regeneration. The glial scar formed by the astrocyte has an initial beneficial effect of walling off the lesion from the intact parenchyma, likely protecting the intact tissue from the invading macrophages and other potentially toxic events. Rapidly expanding astrocytic processes create functional walls surrounding the ischemic core, which extend the time available for marshalling endogenous repair mechanisms, e.g., redirection of blood flow to still salvageable parts of the brain and redirection of neurite sprouting and synapse formation to build new circuitry [77]. The glial scar, also produces inhibitory glycoproteins [78], [79], which reduce neurite outgrowth. Administration of MSCs to rodents reduces the glial scar and also reduces the expression of inhibitory glycoproteins, thereby creating a permissive environment for neurite outgrowth [24], [80]. The tPA/PAI-1 system associated with the reactive astrocyte impacts ischemic damage and regenerative events. Early after stroke, reactive astrocytes secrete abundant factors, such as tPA as well as PAI-1 [24], [81]–[83]. Increasing PAI-1 inhibits tPA activity and subsequently inhibits the tissue damage within the ischemic area [84], [85]. However, during the sub-acute stage after stroke, our data support the hypothesis that the tPA/PAI-1 system of the reactive astrocytes is beneficial, increasing neurite outgrowth. MSCs reduce PAI-1 in reactive astrocytes, which thereby increase tPA activity. This enhanced tPA activity may increase active neurotrophins [48]–[50], [86]. The mechanisms by which MSCs increase PA activity, await further investigation.

There are many factors that contribute to the improvement of neurological function after MSC treatment of stroke [28], [41], [87]–[91]. We propose that astrocytes contribute to the beneficial effects of exogenously administered MSCs in the CNS. Although the most abundant cells in the CNS, astrocytes have been neglected as modulators of brain remodeling and functional recovery. The tPA/PAI-1 system in astrocytes by promoting brain plasticity leading to functional recovery after treatment of stroke with MSCs may provide a new therapeutic target for stroke and CNS diseases.

## Materials and Methods

All experimental procedures were carried out in accordance with the NIH Guide for the Care and Use of Laboratory Animals and approved by the Institutional Animal Care and Use Committee of Henry Ford Hospital.

### Cell Culture

The MSCs employed in our studies are very well characterized and are provided to us by Theradigm, Inc. (Baltimore, MD) and produced in a GMP facility. Briefly, the bone marrow harvested from the hind legs of C57/Bl6 mice (2∼3 m) were prepared, as previously described [31], [92]–[94]. The MSCs are a heterogeneous cell population, and comply with three well established criteria; they 1. are plastic-adherent, 2. express specific surface antigen expression, and 3. in vitro can differentiate into osteoblasts, adipocytes, and chondroblasts [95]. The mouse MSC populations were analyzed for the following surface antigens for phenotypic characterization: CD29 (>90%), CD44 (>80%) and CD105 (>80%) and were free of hematopoietic cell phenotype CD11b (<1%), CD34 (<1%), and CD45 (<1%). MSCs were cultured with α-modified MEM medium (Hyclone, Logan, UT) containing 20% fetal bovine serum (FBS, Gibco Laboratory, Grand Island, NY) and penicillin-streptomycin on 75 cm^2^ tissue culture flasks (Corning St. Louis, MO). Mouse cortical astrocytes, C8-D1A (Astrocyte type I clone from C57/BL6 strains), were obtained from the American Type Culture Collection (ATCC, CRL-2541™, Arlington, VA). Cells were cultured in high glucose Dulbecco's modified eagle medium (DMEM, Invitrogen, San Diego, CA) with 10% FBS, containing penicillin-streptomycin on 75 cm^2^ tissue culture flasks, and all the cells were placed in an moist incubator and cultured at 37°C, with 5% CO_2_.

### OGD Treatment of Astrocytes

Astrocytes (1×10^5^) were seeded in each well of a 6-well plate containing normal medium. After cells grew to 70% confluence, the medium was replaced with non-glucose culture media and cultured in an anaerobic chamber (model 1025, Forma Scientific, OH) for 2 hrs. The astrocytes were then cultured under normal conditions with or without MSCs. For co-culture with MSCs, an upper chamber of the transwell insert dish (Becton Dickinson Labware, FALCON®) was used with a ratio 1∶100 of co-cultured MSCs to astrocytes. Astrocytes with or without MSC co-culture were detached and collected for RNA and protein extraction after 24 hrs.

### Preparation of Conditioned Media

To prepare the conditioned media for the primary cortical neuronal culture, Gibco™ Neurobasal™ Medium (Invitrogen, Cat No. 21103) supplemented with B27 (Invitrogen, Cat No. 17504-044) and L-Glutamine-Penicillin-Streptomycin solution (Sigma-Aldrich, Cat No. G6784) were used as basal culture media. Briefly, following 2 hrs of OGD and subsequently normal culture conditions with or without MSCs co-cultured for 24hrs, normal and OGD astrocytes, respectively, were rinsed with basal culture media. These astrocytes were then cultured in the basal culture media for an additional 12 hrs with MSCs omitted. Media from the various treatment groups are referred to as conditioned media. Conditioned media were filtered with a 0.22µm-pore filter and stored in -80°C for further studies. The conditioned media were harvested from the replaced fresh basal culture media; therefore, the tPA activity in the conditioned media derive solely from the cultured astrocytes.

To knock-down the tPA expression in astrocytes, siRNA technique was employed. Following the protocol provided by the company, tPA siRNA (M-048467-01-0005, Thermo Fisher Scientific. Lafayette CO 80026) and negative control siRNA were transfected into triple well cultured astrocytes seeded in six well plates, respectively. Conditioned media from these astrocytes as well as normal cultured astrocytes were made following the procedure described above, and the astrocytes in individual wells were lysed and for Western blot analysis.

### ELISA Detection of the Total tPA and Active tPA

A mouse tPA total antigen assay kit (Innovative research, Catalog No. MTPAKT-TOT) and a mouse tPA activity assay kit (Innovative research, Catalog No. MTPAKT) were used to detect the total tPA protein and active tPA level in conditioned media, respectively. Following the manufacturer's assay procedures, conditioned media from normal and OGD astrocytes with or without MSC co-culture were added into the wells of ELISA plates, as well as various diluted tPA standards. The primary antibody and second antibody were sequentially added. The reaction was quenched by the addition of 1M H_2_SO_4_, The absorbance values were read at 450nm.

### MCAo and Tissue Preparation

Adult male mice (C57/BL6 strains, 2 months old, weighing 25–29g) were employed in our study. Briefly, mice were initially anesthetized with 3.5% isoflurane and maintained with 1.0% to 2.0% isoflurane in 70% N_2_O and 30% O_2_ using a facemask. The rectal temperature was controlled at 37°C with a feedback-regulated water heating system. The right common carotid artery, external carotid artery (ECA), and internal carotid artery (ICA) were exposed. A length of 6-0 monofilament nylon suture (8.0–9.0 mm), determined by the animal weight, with its tip rounded by heating near a flame, was advanced from the ECA into the lumen of the ICA until it blocked the origin of the MCA. At 1 day post-ischemia, randomly selected mice (n = 9) received MSC transplantation.

For MSC transplantation, mice were initially anesthetized with 3.5% Isoflurane and maintained with 1.5% Isoflurane in 70% N_2_O and 30% O_2_ using a face mask. Approximately 1×10^6^ MSCs in 0.2 ml total fluid volume of phosphate-buffered saline (PBS) were injected into a tail vein. Immunosuppressants were not used in any animal. Mice injected with PBS alone were employed as MCAo control (n = 9). A third group of naive mice without surgery and treatment was employed as normal controls (n = 9).

All animals were sacrificed under deep ketamine anesthesia at 14 days after MCAo, among which 9 mice (n = 3 for normal control, MCAo alone and with MSC treatment, respectively) were employed for tissue protein extraction, which were used for Western blot and direct casein zymography. The remaining 18 mice (n = 6 for normal control, MCAo alone and with MSC treatment, respectively) were perfused with 0.9% saline, and a series of frozen brain coronal sections (8 µm) were obtained for histochemistry staining and Laser Capture Microdissection (LCM).

### Protein and RNA Isolation

Brain tissues from mice (n = 3/group) along the IBZ ipsilateral to the injury were extracted. These brain tissues were homogenized, and were used to isolate total RNA and protein with TRIzol (Invitrogen, San Diego, CA), following a standard protocol.

The astrocytes cultured under various conditions were harvested and rinsed with PBS, then lysed in the RIPA lysis buffer containing proteinase inhibitor cocktail (Roche, Indianapolis, IN). Protein concentrations were determined using the Bicinchoninic Acid (BCA) protocol (Pierce, Rockford, IL), loaded on 10% Bis-Tris Gels (Invitrogen, San Diego, CA), and then processed for Western blotting.

### LCM Isolation of Reactive Astrocytes in the IBZ

Cryostat sections stored at −80°C were immediately immersed in acetone for 2 min fixation and air-dried for 30 sec. After a brief rinse with 0.1% diethylpyrocarbonate treated phosphate-buffered saline (PBS), sections were incubated with GFAP antibody (Dako Z0334; Dako, Carpinteria, CA) at 1∶50 dilution for 5 min, rinsed with PBS twice, and then incubated with 1∶100 dilution CY3-conjugated F(ab′)_2_ anti-rabbit IgG secondary antibody for 5 min. rinsed with PBS twice and air-drying for 5 min, GFAP positive reactive astrocytes along the ischemic boundary were cut using Leica LMD6000 system. All reaction steps were performed in RNase-free solutions. Approximately 5,000 cells were dissected and collected in Eppendorf tubes containing 100 µL of lysis buffer. The samples were stored in −80°C before RNA isolation.

### Quantitative Real Time PCR (qRT-PCR)

qRT-PCR was performed with the isolated total RNA transcribed into cDNA using poly-dT oligonucleotides following the manufacturer's instructions. Quantitative PCR for total cDNAs was performed in the ABI PRISM 7000 Sequence Detection System, using the standard protocols with the Quantitec SYBY Green PCR Kit (Qiagen, Valencia, CA). The following primers were purchased from Invitrogen: mouse tPA, forward: CTGAGGTCACAGTCCAAGCA, reverse: ACAGATGCTGTGAGGTGCAG; mouse PAI-1, forward: GTCTTTCCGACCAAGAGCAG, reverse: ATCACTTGGCCCATGAAGAG; and mouse GAPDH, forward GTCTACTGGTGTCTTCACCACCAT, reverse: GTTGTCATATTTCTCGTGGTTCAC. GAPDH was used as an internal control for gene expression.

### Western Blot Assay

The total protein was used for Western blot assay following the standard Western blotting protocol (Molecular Clone, Edition II). The concentrations of the primary antibodies employed were: tPA (1∶2000, Santa Cruz, sc-15346), PAI-1 (1∶2000, Santa Cruz, sc-8979), and beta actin (1∶5000, Santa Cruz, sc-1616). Respective horseradish peroxidase (HRP) labeled secondary antibodies were applied and enhanced chemiluminescence (ECL) detection was used according to the manufacturer's instructions (Pierce, Rockford, IL). The integrated density mean grey value of the band was analyzed under ImageJ software and the corresponding relative expression ratio was calculated.

### Direct Casein Zymography for tPA Activity

Proteins from culture supernatant, cells and brain tissues were separated by 10% SDS-PAGE and tPA activity was assayed by zymography, as detailed previously [96]. Briefly, 10µg protein samples or 30µL conditioned supernatant were mixed with the sample loading buffer without β-ME, and heating was omitted. The mixture of the lower gel (10% acrylamide) contained casein (1 mg/ml, Sigma) and plasminogen (13 mg/ml, American Diagnostica, Greenwich, CT) as substrates for plasmin and PA, respectively. The gel was then washed for 30 min with 2.5% Triton X-100 to remove SDS and further washed for 10 min with 0.1 M Tris buffer, pH 8. The new Tris buffer was replaced and the gel was incubated for 4 hrs at 37°C to allow caseinolysis occur. On the darkly stained casein background, PA activity was visualized as light bands resulting from casein degradation. To verify loading variations, duplicate samples were used in gel electrophoresis. After electrophoresis, the gel was stained with Coomassie Blue R-250 and destained with 40% methanol as well as 10% acetic acid.

### Cortical Cell Primary Culture

The cortices were dissected from 17–18 day embryos of C57/BL6 mice and dissociated in Ca^2+^- and Mg^2+^- free Hanks balance salt solution (HBSS) containing 0.125% trypsin for 30 min. Cells were washed with DMEM containing 5% FBS, triturated in DMEM/5% FBS and passed through the cell strainer (BD Falcon REF 352350). The cell density was determined by a hemacytometer and 2×10^4^ cells per well were plated on eight-well chamber slides (Nalge Nunc International, Naperville, IL). The cells were incubated at 37°C with 5% CO_2_ overnight and switched to the serum-free Neurobasal medium with B27 supplement for 3 days, and the medium was then changed to conditioned media. Treatment groups include: 1) neural basal medium as control, 2) medium from normal cultured astrocytes, 3) medium from normal cultured astrocytes co-cultured with MSCs, 4) medium from normal cultured astrocytes co-cultured with MSCs and 200ng/mL tPA neutralizing antibody (Product No. 1188, American Diagnostica Inc., Stamford, CT) [97]–[99] was added, 5) medium from OGD astrocytes, 6) medium from OGD astrocytes co-cultured with MSCs, 7) medium from OGD astrocytes co-cultured with MSCs and 200ng/mL tPA neutralizing antibody was added, 8) 15nM rh-tPA (Genentech Inc., South San Francisco, CA), 9) medium from astrocytes transfected with negative control siRNA, and 10) medium from astrocytes transfected with tPA siRNA.

### Neurite Outgrowth Assay

After 4 days in culture, neural cells were fixed with 4% paraformaldehyde and stained with immunofluorescence for beta-tubulin (Tuj 1) identification. To analyze neurite outgrowth, TuJ1-positive cells were digitized using a 20× objective (Zeiss) via the MicroComputer Imaging Device (MCID) analysis system (Imaging Research, St. Catharines, Ontario, Canada). Neurite outgrowth was quantified using a software program developed in our laboratory that includes measurements of the number and length of branches [100]. At least fifty TuJ1-positive cells, distributed in 9 random fields per well and triple wells per group, were measured, all measurements were performed by experimenters blinded to each culture condition.

### Histochemistry and Immunostaining

The axonal fibers of the mouse striatum were examined using a combined Nissl- and silver-staining method (Bielshowsky staining) [101]. Double staining for Bielshowsky and Luxol fast blue [26] was used to demonstrate axons and myelin, respectively. Briefly, for Bielshowsky staining, frozen brain slides centered at the ischemic core (coordinates bregma −0.5∼0.5 mm) [102] were placed in 20% silver nitrate in the dark, then ammonium hydroxide was added to stain the slides until the tissues turned brown with a gold background and they were then treated with sodium thiosulfate. Slides were then stained in Luxol fast blue solution, washed in 95% alcohol, and subsequently placed in lithium carbonate. Nuclei should be colorless; myelin should be blue, and axons should appear black. Sections were analyzed with an optical microscope and pictures were obtained along the IBZ of the striatum.

Frozen brain sections were incubated with the primary antibody against synaptophysin (1∶100, 60min, RT, Chemicon, MAB5258), followed with Cy3 labeled secondary antibody. Sections were observed with a fluorescence microscope and pictures were taken along the IBZ.

MCID software was used to analyze the integrated density of labeled axonal fibers and synaptophysin in the IBZ. Five randomly selected areas along the IBZ were analyzed per animal.

To identify the neuronal apoptosis in the IBZ, double-staining with an ApopTag® Fluorescein In Situ Apoptosis Detection Kit (Millipore, Cat # S7110) and with antibody against microtubule-associated protein 2 (1∶200, 4°C overnight, Chemicon, MAB3418) was employed to stain the frozen brain sections. Sections were evaluated using a fluorescence microscope and pictures were taken along the IBZ. The double stained cells in eight randomly selected areas along the IBZ were measured per section and 3 sections were taken per animal.

### Statistics

Data are expressed as means±SE. The differences between mean values were evaluated with the two tailed Student's t-test (for 2 groups) and the analysis of variance (ANOVA, for >2 groups). All calculations and statistical tests were performed by the computer programs Microsoft Excel 2000 (Microsoft, Redmond, WA) or SPSS 11.5 (SPSS, Chicago, IL). P<0.05 was considered significant for all analyses.
